# Microbial dysbiosis in obstructive sleep apnea: a systematic review and meta-analysis

**DOI:** 10.3389/fmicb.2025.1572637

**Published:** 2025-05-15

**Authors:** Yang Guo, Shuqi Sun, Yaoyao Wang, Shiyang Chen, Ziwei Kou, Peng Yuan, Wei Han, Xinjuan Yu

**Affiliations:** ^1^School of Medical Laboratory, Shandong Second Medical University, Weifang, China; ^2^School of Clinical Medicine, Shandong Second Medical University, Weifang, China; ^3^Department of Medicine, Qingdao University, Qingdao, China; ^4^Qingdao Key Laboratory of Common Diseases, Department of Respiratory and Critical Medicine, Department of Emergency, Department of General Practice, Qingdao Municipal Hospital, University of Health and Rehabilitation Sciences, Qingdao, China; ^5^Clinical Research Center, Qingdao Key Laboratory of Common Diseases, Qingdao Municipal Hospital, University of Health and Rehabilitation Sciences, Qingdao, China

**Keywords:** obstructive sleep apnea, gut microbiota, oral microbiota, respiratory tracts microbiota, systematic review, meta-analysis

## Abstract

**Background:**

The association between the microbiota and obstructive sleep apnea (OSA) remains understudied. In this study, we conducted a comprehensive systematic review and meta-analysis of studies investigating the diversity and relative abundance of microbiota in the gut, respiratory tracts and oral cavity of patients with OSA, aiming to provide an in-depth characterization of the microbial communities associated with OSA.

**Methods:**

A comprehensive literature search across PubMed, the Cochrane Library, Web of Science, and Embase databases were conducted to include studies published prior to Dec 2024 that compared the gut, respiratory and oral microbiota between individuals with and without OSA. The findings regarding alpha-diversity, beta-diversity, and relative abundance of microbiota extracted from the included studies were summarized. This meta-analysis was conducted in accordance with the Preferred Reporting Items for Systematic Reviews and Meta-Analyses (PRISMA) guidelines, and the study protocol was registered with PROSPERO (CRD42024525114).

**Results:**

We identified a total of 753 articles, out of which 27 studies were ultimately included in the systematic review, involving 1,381 patients with OSA and 692 non-OSA populations, including 1,215 OSA patients and 537 non-OSA populations in adults and 166 OSA patients and 155 non-OSA populations in children. The results of alpha diversity revealed a reduction in the Chao1 index (SMD = −0.40, 95% CI = −0.76 to −0.05), Observed species (SMD = −0.50, 95% CI = −0.89 to −0.12) and Shannon index (SMD = −0.27, 95% CI = −0.47 to −0.08) of the gut microbiota in patients with OSA. Beta diversity analysis indicated significant differences in the gut, respiratory and oral microbial community structure between individuals with OSA and those without in more than half of the included studies. Furthermore, in comparison to the non-OSA individuals, the gut environment of patients with OSA exhibited an increased relative abundance of phylum Firmicutes, along with elevated levels of genera *Lachnospira*; conversely, there was a decreased relative abundance of phylum Bacteroidetes and genus *Ruminococcus* and *Faecalibacterium*. Similarly, within the oral environment of OSA patients, there was an elevated relative abundance of phylum Actinobacteria and genera *Neisseria*, *Rothia*, and *Actinomyces*.

**Conclusion:**

Patients with OSA exhibit reduced diversity, changes in bacterial abundance, and altered structure in the microbiota, especially in the gut microbiota. The results of this study provide basic evidence for further exploration of microbiome diagnostic markers and potential intervention strategies for OSA.

## 1 Introduction

Obstructive sleep apnea (OSA) is a multifaceted disorder characterized by partial or complete obstruction of the upper airway during sleep, leading to disrupted sleep architecture and intermittent hypoxia, with hypercapnia as the primary underlying pathophysiological mechanism (Li et al., 2023). The prevalence of OSA in the general population in adult ranges from 9 to 38% (Senaratna et al., 2017). Furthermore, OSA has been linked with various cardiovascular, metabolic, and cognitive disorders (McNicholas et al., 2018; Ryan et al., 2020). The prevalence of OSA in patients with hypertension, heart failure, coronary artery disease, pulmonary hypertension, atrial fibrillation, and stroke is reported to range from 40 to 80% (Javaheri et al., 2017). Neurohormonal dysregulation, metabolic abnormalities, systemic inflammation, and enhanced oxidative stress are potential mechanisms through which OSA contributes to increased risk of all-cause mortality and cardiovascular mortality (Yeghiazarians et al., 2021; Chen et al., 2021).

Physiologically, the microbiota and their metabolites play a crucial role in maintaining homeostasis of the host’s metabolic, immune, and neuroendocrine systems (Kho and Lal, 2018). With advancements in molecular tools and technologies such as 16S rRNA high-throughput sequencing and metagenomics, the significance of microbiota as a pivotal determinant for OSA is progressively unraveling (de Vos et al., 2022).

Gut microbiota diversity is a crucial indicator of health, and reduced *α*-diversity may be deemed detrimental to the host due to the proliferation of pathogenic microorganisms (Le Chatelier et al., 2013). However, it remains controversial whether altered microbial diversity serves as one of the risk factors for OSA owing to differences in design, sample size, and sites of the studies. Valentini et al. (2020) demonstrated diminished gut microbiota diversity in children with OSA. A recent study from Chen et al. reported that the OSA group had a significantly lower salivary microbial richness than the controls (Chen et al., 2021). In contrast, Ko et al. (2019) did not observe any disparities in alpha diversity in their investigation on OSA and its oral microbial diversity. Wu and coworkers (Wu et al., 2022) found that there was no significant difference in alpha diversity of gut microbiota between patients with OSA and the controls. In addition, the changes in bacterial abundance are inconsistent in different studies. Previous experiments have found that the phylum Firmicutes increased, and Bacteroidetes decreased in the gut microbiota of patients with OSA (Lu et al., 2022; Wang et al., 2022; Zhang et al., 2022). While, Li et al. (2023) found that there was no significant difference in Firmicutes, Bacteroides, Actinobacteria, and Proteobacteria between patients with OSA and the controls after comparing the gut samples. After comparing the salivary samples of OSA patients with healthy controls, a recent study found *Prevotella*, *Actinomyces*, *Bifidobacterium*, *Escherichia* and *Lactobacillus* were enriched in the OSA group (Huang et al., 2022). However, another report revealed that the relative abundances of *Prevotella*, *Veillonella*, *Bacteroides*, *Alloprevotella* and *Leptotrichia* in the oral microbiota of patients with severe OSA were significantly lower than those in the healthy controls (Gao et al., 2023).

The consistency of microbiota alterations in patients with OSA remains a subject of debate. Thus, we conducted a meta-analysis to evaluate the reproducibility and specificity of microbiota alterations in 1381 patients with OSA and to investigate the role and potential mechanisms of dysbiosis in OSA.

## 2 Materials and methods

This meta-analysis was conducted in accordance with the Preferred Reporting Items for Systematic Reviews and Meta-Analyses (PRISMA) guidelines (Moher et al., 2009), and the study protocol was registered with PROSPERO (CRD42024525114).

### 2.1 Search strategy

**Figure 1:**
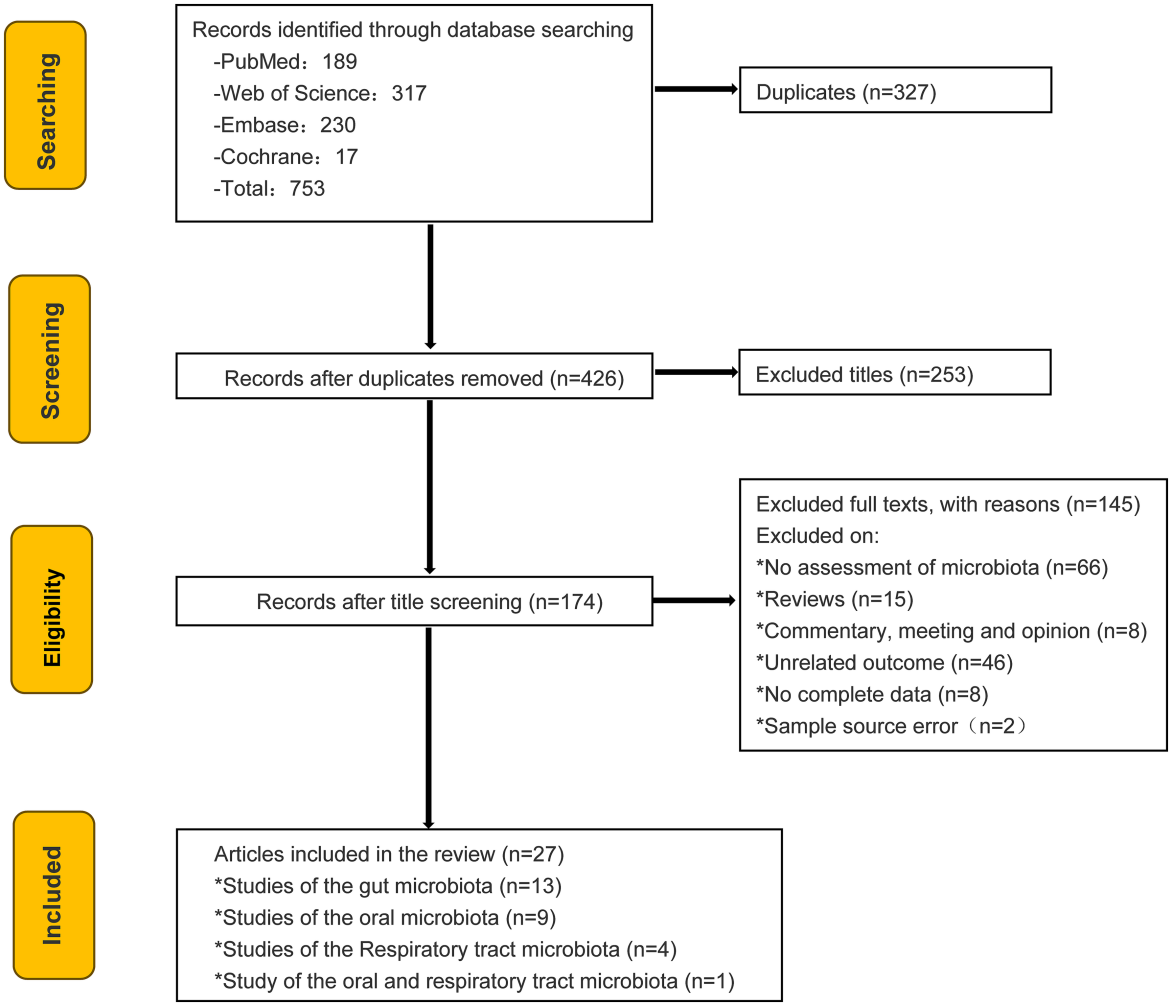
PRISMA flow diagram showing the study selection process.

Two authors independently conducted comprehensive literature searches in PubMed, The Cochrane Library, Web of Science, and Embase using the search strategies outlined in Supplementary Table 1. These literatures were last updated in Dec. 2024. To ensure the comprehensiveness of the literature search, a secondary search strategy was implemented. A “reverse snowballing” approach was employed to identify relevant studies from the reference lists of included studies that were not captured by the initial search equation. The final selection was agreed upon by all authors. The selection process of the included studies is shown in Figure 1.

### 2.2 Study selection and quality evaluation

The inclusion criteria were as follows: (1) the study design is an observational case–control or cross sectional study published in English or Chinese; (2) the study population consists of human subjects with OSA of all ages; (3) the OSA group were patients with AHI ≥ 5 under PSG or OCST monitoring, and all patients had not received systemic treatment before the experiment; (4) the article addresses one or more of the following microbiota characteristics: *α*-diversity index, *β*-diversity, and relative abundance of bacteria.

The exclusion criteria were as follows: case reports, reviews, and review studies; duplicate studies; studies from which appropriate data could not be extracted or contained data errors; studies unrelated to the subject matter; and studies involving animal testing.

### 2.3 Data extraction and methodological assessment

Two researchers (Guo and Sun) independently conducted a thorough screening of the eligible studies, excluding studies that did not meet the predefined inclusion criteria. To ensure consistent screening criteria, two investigators underwent standardized training prior to conducting the formal literature screening. The following data were meticulously collected: study characteristics including first author, year of publication, and country of study; population characteristics encompassing sample size of cases and controls, as well as age and gender distribution; sample characteristics such as type of sample and microbiota assay method employed; comprehensive assessment of microbiota characteristics comprising *α* diversity measures like Chao 1 index, Observed species count, Shannon index, Simpson index; *β* diversity analysis; relative abundance at both phylum and genus levels. Methodological evaluation of the included studies was performed using the Newcastle-Ottawa Scale (NOS) (Stang, 2010). The NOS assigned scores based on three key aspects of the included studies: selection, comparability, and outcome. In this assessment process, the selection section had a maximum score of 4 points, comparability had 2 points, and outcome had 3 points, resulting in a total possible score of 9 points. The studies were considered to be high quality if they achieved a maximum score of 9 and a total score of ≥ 5. Two researchers independently evaluated the scores, and any discrepancies were resolved through consensus.

### 2.4 Data analysis

Microbiota diversity and relative abundance differences among groups were assessed using GraphPad Prism 9 software, with effect sizes for continuous variables reported as standardized mean difference (SMD). The results of *α*-diversity were visualized through the creation of forest plots using Review manager 5.3 software. For studies reporting only median and interquartile range, we employed the median and previously established formulas to estimate the mean and standard deviation (Hozo et al., 2005). The heterogeneity was assessed using I^2^ values, with an I^2^ value of 25% considered as low heterogeneity, 50% as moderate heterogeneity, and 75% as high heterogeneity. Sensitivity analyses were conducted by systematically removing one study at a time to evaluate the robustness of the results. Publication bias was evaluated using the Begg test and Egger’s regression asymmetry test. Statistical significance was defined as a two-sided *p* value less than 0.05.

## 3 Results

### 3.1 Search results and study eligibility

According to the search terms, a total of 753 articles on English literature were initially retrieved. Out of these, 173 articles underwent screening based on their titles and abstracts. Following a comprehensive examination of the complete text, we excluded literature that featured interventions incompatible with our study, endpoints lacking microbiota count information, as well as incomplete or incorrect data. Consequently, we identified and selected 27 studies for further analysis: 13 pertaining to gut microbiota, 9 focusing on oral microbiota, 4 addressing respiratory microbiota, and 1 addressing both oral and respiratory microbiota. Among the articles focusing on oral microbiota, 7 studies were performed using 16S rRNA sequencing on the Illumina platform, primarily utilizing the MiSeq platform, 2 studies utilized the whole-genome metagenomic sequencing. The study samples for these articles consisted of 4 saliva, 4 oral swabs, and 1 oral rinse. The gut and respiratory tract microbiota all were performed using 16S rRNA sequencing on the Illumina platform, utilizing stools as the gut microbiota sample source. 2 nasal swabs, 1 nasal 1avage and 1 bronchoalveolar lavage fluid (BALF) as respiratory tract sample types. The NOS scores were 6–9, and all studies were high quality studies. The characteristics of the studies included in the meta-analysis were presented in Table 1.

**Table 1:** Characteristics of the included studies.

Study year	Country	Setting	Sample Type	Sample collection time	Age (case/control)	BMI (case/control)	Sample size (case/control)	Gender (case/control)	Severity of OSA (M/Mod/S)	Microbiota assessment	Score
Ko et al. (2019)	China	Lab	Stool	Morning	45.8 ± 11.8/39.0 ± 8.9	27.4 ± 4.67/24.3 ± 2.25	93/20	Case (M 80/F 12) Control (M 11/F 9)	40/23/30	16S rRNA sequencing (V3–V4) on the Illumina MiSeq system	6
Valentini et al. (2020)	Italy	Hospital	Stool	NR	5.0 ± 1.9/8.7 ± 3.6	16.8 ± 2.5/17.9 ± 3.9	7/8	Case (M 6/F 1) Control (M 4/F 4)	NR	16S rRNA sequencing (V3–V4) on the Illumina MiSeq system	6
Wang et al. (2021)	China	Hospital	Stool	Morning	75.26 ± 7.14/49.22 ± 3.26	27.8 ± 1.39/24.7 ± 0.40	100/27	Case (M 79/F 21) Control (M 19/F 8)	23/17/60	16S rRNA sequencing (V3–V4) on the Illumina NovaSeq platform	8
Wu et al. (2022)	China	Community	Stool	NR	5.08 ± 0.53/4.91 ± 0.56	16.24 ± 2.10/15.78 ± 1.75	43/45	Case (M 21/F 22) Control (M 27/F 18)	NR	16S rRNA sequencing (V4) on the Illumina MiSeq system	8
Wang et al. (2022)	China	Hospital	Stool	NR	38.75 ± 10.40/42.29 ± 10.26	23.86 ± 3.12/22.26 ± 3.54	32/14	Case (M 30/F 2) Control (M 10/F 4)	NR	16S rRNA sequencing (V3–V4) on the Illumina MiSeq PE300 system	7
Bikov et al. (2022)	England	Community	Stool	NR	55 ± 12/43 ± 16	26.3 (25.0–28.1)/22.8 (20.9–27.1)	19/20	Case (M 10/F 9) Control (M 7/F 13)	NR	16S rRNA sequencing (V3–V4) on the Illumina MiSeq system	8
Li et al. (2023)	China	NM	Stool	Morning	37 (30–51)/38 (33–40)	27.35 ± 2.97/24.01 ± 3.11	37/11	Case (M 30/F 7) Control (M 7/F 4)	11/11/15	16S rRNA sequencing (V3–V4) on the Illumina MiSeq PE300 system	7
Zhang et al. (2022)	China	Lab	Stool	NR	50.2 ± 10.3/44.6 ± 18.1	29.5 ± 4.9/26.6 ± 5.3	38/9	Case (M 31/F 7) Control (M 8/F 1)	NR	16S rRNA sequencing (V3–V4) on an Ion S5TM XL platform	8
Zhu et al. (2024)	China	Hospital	Stool	Morning	44.82 ± 10.67/44.82 ± 10.67	29.43 ± 3.53/21.53 ± 2.32	39/20	Case (M 32/F 7) Control (M 15/F 5)	NR	16S rRNA sequencing (V3–V4) on the Illumina MiSeq system	7
Guo et al. (2024)	China	Hospital	Stool	Morning	40.21 ± 10.99/33.06 ± 5.86	26.32 ± 3.62/22.51 ± 3.32	97/16	Case (M 84/F 13) Control (M 12/F 4)	NR	16S rRNA sequencing (V3–V4) on the Illumina MiSeq system	7
Liu et al. (2024)	China	Hospital	Stool	morning	40.21 ± 10.99/42.20 ± 14.77	24.27 ± 2.9/24.60 ± 2.73	27/10	Case (M 18/F 9) Control (M 5/F 5)	10/NR/NR	Whole-genome metagenomic	6
Wang et al. (2024)	China	Hospital	Stool	Morning	53.86 ± 16.91/41.74 ± 16.1	26.67 ± 3.00/24.74 ± 4.91	45/19	Case (M 30/F 15) Control (M 13/F 6)	14/13/18	16S rRNA sequencing (V3–V4) on the Illumina MiSeq system	7
Xue et al. (2024)	China	Hospital	Stool	NR	44.5 ± 12.0/39.1 ± 14.0	31.8 ± 7.0/21.1 ± 2.2	12/7	Case (M 10/F 2) Control (M 3/F 7)	NR	Whole-genome metagenomic	7
Xu et al. (2018)	China	Hospital	Oral swab	Morning	6 (5–8)/6 (6–8)	1.7 ± 0.8/1.6 ± 0.3	30/30	Case (M 22/F 8) Control (M 23/F 7)	NR	Whole-genome metagenomic	7
Ko et al. (2019)	China	Hospital	Oral swab	Morning	45.80 ± 13.05/35.92 ± 7.69	NR/NR	126/13	Case (M 112/F 14) Control (M 9/F 4)	35/NR/NR	16S rRNA sequencing (V3–V4) on the Illumina MiSeq system	8
Yang et al. (2019)	China	Hospital	Oral swab	NR	40.3 ± 10.8/40.3 ± 10.8	27.8 ± 3.2/27.3 ± 3.3	26/25	NR	NR	16S rRNA sequencing on the Illumina platform	6
Jia et al. (2020)	China	Hospital	Saliva	Morning	47.0 ± 9.5/40.2 ± 9.4	27.0 ± 3.8/28.5 ± 6.4	15/9	Case (M 13/F 2) Control (M 6/F 3)	NR	16S rRNA sequencing (V3–V4) on the Illumina MiSeq PE250 system	7
Chen et al. (2021)	China	Hospital	Saliva	Morning	27.9 ± 3.2/29.3 ± 2.8	25.6 ± 2.3/23.2 ± 2.4	27/27	Case NR Control NR	NR	16S rRNA sequencing (V3–V4) on the Illumina NovaSeqTM 6,000 platform	7
Huang et al. (2022)	China	Hospital	Saliva	Morning	7.47 ± 2.24/7.55 ± 2.48	17.4 (15.0, 22.1)/16.5 (14.5, 18.1)	36/22	Case (M 17/F 19) Control (M 9/F 13)	NR	16S rRNA sequencing (V3–V4) on the Illumina NovaSeq platform	8
Chen et al. (2022)	China	Hospital	Saliva	Morning	40.12 ± 10.39/30.50 ± 5.74	27.1 ± 2.61/24.60 ± 3.08	53/27	Case NR Control NR	NR	16S rRNA sequencing (V3–V4) on the Illumina NovaSeq TM 6000 platform	6
Gao et al. (2023)	China	Hospital	Oral rinse	Morning	75.26 ± 7.14/45.6 ± 12.4	30.9 ± 6.7/21.1 ± 2.2	7/7	Case (M 6/F 1) Control (M 3/F 4)	NR	Whole-genome metagenomic	8
Zhu and Teng (2024)	China	Hospital	Oral swab	NR	5.11 ± 1.69/5.32 ± 2.49	NR/NR	20/20	Case (M 15/F 5) Control (M 16/F 4)	NR	16S rRNA sequencing on the Illumina NovaSeq 600 platform	7
Zhang et al. (2023)	China	Hospital	Oral swab and Nose swab	Morning	7 (5–8)/6 (3–10)	15.2 (14.0–17.0)/16.6 (14.7–17.9)	30/30	Case (M 16/F 14) Control (M 16/F 14)	NR	16S rRNA sequencing (V3–V4) on the Illumina NovaSeq platform	7
Lu et al. (2018)	China	Hospital	BALF	NR	48.6 ± 1.8/49.0 ± 2.3	28.2 ± 0.5/26.0 ± 0.6	11/8	NR	NR	16S rRNA sequencing (V4–V5) on the Illumina platform	7
Wu et al. (2019) discover cohort	America	Community	Nasal lavage	NR	54 (47.0–61.2)/49 (44.0–54.0)	29.9 (26.4, 37.3)/27.5 (24.6, 30.0)	304/168	Case (M 270/F 34) Control (M 119/F 49)	172/87/45	16S rRNA sequencing (V4) on the Illumina MiSeq system	7
Wu et al. (2019) validation cohort	America	Community	Nasal lavage	NR	46 (34.7–58.5)/41 (31.7–54.5)	28.9 (24.5, 31.7)/27.0 (25.0, 29.5)	68/25	Case (M 57/F 11) Control (M14/F 11)	19/18/31	16S rRNA sequencing (V4) on the Illumina MiSeq system	7
Hong et al. (2022)	Korea	Community	Nasal swab	NR	64.02 ± 6.63/61.03 ± 4.04	25.28 ± 2.87/23.82 ± 2.66	54/38	Case (M 24/F 30) Control (M 15/F 23)	NR	16S rRNA sequencing (V3–V4) on the Illumina MiSeq system	7
Lenk et al. (2023)	Germany	Hospital	Nasal swab	NR	52 (43–63.5)/58 (49.5–62.5)	31.11 (25.56, 39.97)/25.71 (24.03, 30.74)	22/17	Case (M 18/F4) Control (M 8/F 9)	NR	16S rRNA sequencing (V3–V4) on the Illumina MiSeq system	8

NR, not reported; BALF, bronchoalveolar lavage fluid.

When characterizing the microbial attributes of various anatomical regions in the human body, including the oral cavity, airway, and gut, researchers predominantly employ alpha diversity indices. Analysis of aggregated data reveals significant variations in alpha diversity indices across different study populations (Kou et al., 2024). However, notable heterogeneity exists within studies conducted on the same population with regards to alpha diversity indices, which can be attributed to factors such as sample size, types of samples collected, and sampling procedures (Supplementary Tables 2–4).

### 3.2 Alpha diversity

**Figure 2:**
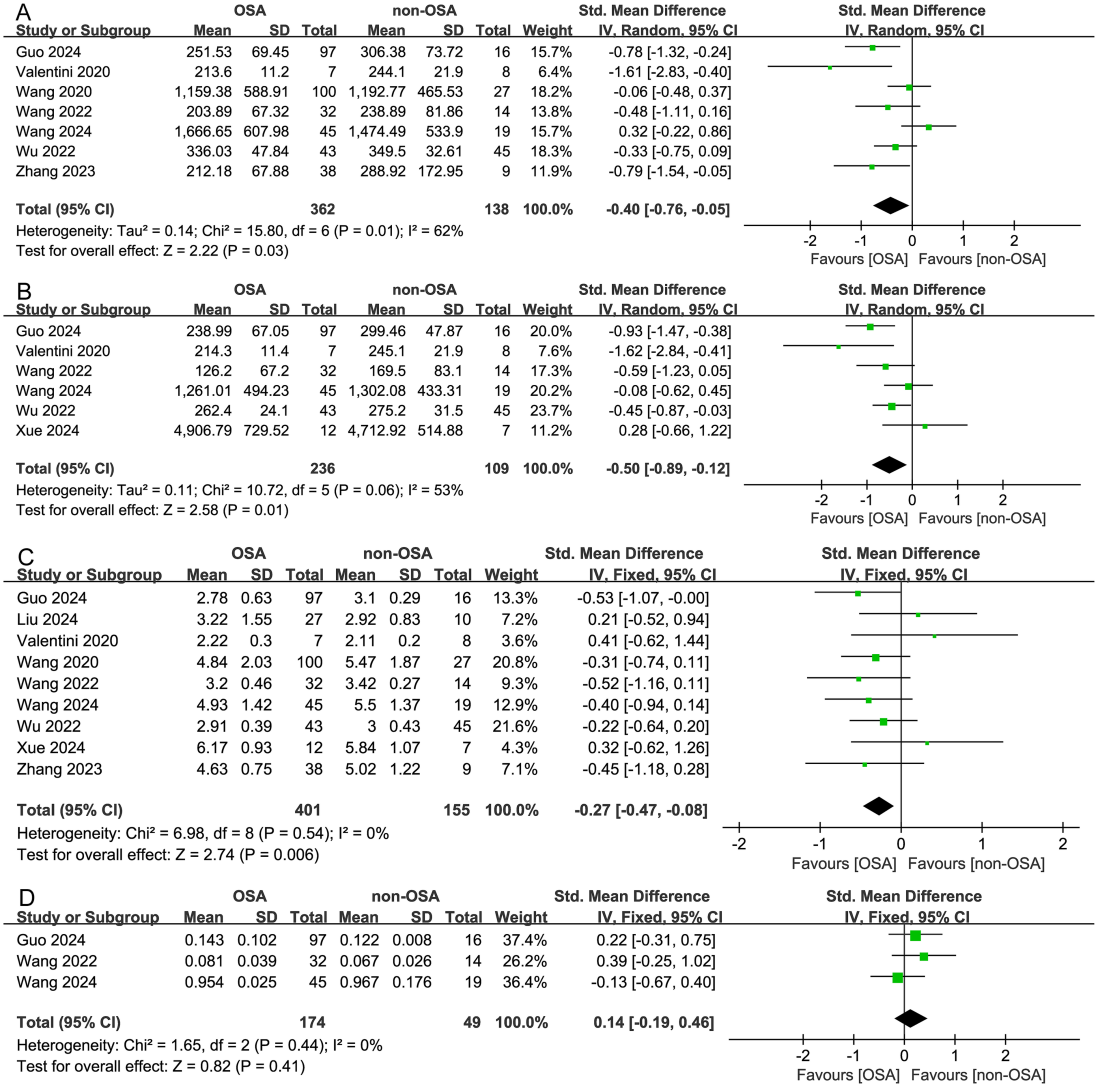
Forest plot of comparing gut microbiota alpha diversity in OSA and non-OSA populations. **(A)** Chao1 index; **(B)** Observed species; **(C)** Shannon index; **(D)** Simpson index.

Among the 13 studies focusing on gut microbiota, *α* diversity was reported in 9 of them. Based on the forest plot, OSA patients exhibited significantly reduced microbial *α* diversity compared to controls. The assessed indices included Chao1 index, Observed species, Shannon index, and Simpson index. The Chao1 index (SMD = −0.40, 95% CI = −0.76 to −0.05, *I^2^* = 62%, *p* = 0.01, *n* = 7) with moderate heterogeneity across studies, Observed species (SMD = −0.50, 95% CI = −0.89 to −0.12, *I^2^* = 53%, *p* = 0.06, *n* = 6) with moderate heterogeneity across studies, Shannon index (SMD = −0.27, 95% CI = −0.47 to −0.08, *I^2^* = 0%, *p* = 0.54, *n* = 9) of the OSA patients were significantly lower than those of non-OSA individuals (Figures 2A–C). However, the Simpson index was no differ significantly between OSA and non-OSA (SMD = 0.14, 95% CI = −0.19 to 0.46; *I^2^* = 0%, *p* = 0.44, *n* = 3) (Figure 2D). In subgroup analyses of the population, the adult OSA group exhibited lower *α* diversity compared to the control group [the Chao1 index (SMD = −0.32, 95% CI = −0.75 to 0.10. *I^2^* = 65%, *p* = 0.02, *n* = 5), Shannon index (SMD = −0.32, 95% CI = −0.55 to −0.10; *I^2^* = 0%, *p* = 0.54, *n* = 7) (Supplementary Figures 1A,B)]. While no significant differences in α diversity were observed between the pediatric OSA group and non-OSA group [the Chao1 index (SMD = −0.84, 95% CI = −2.07 to 0.40. *I^2^* = 74%, *p* = 0.05, *n* = 2), Shannon index (SMD = −0.13; 95% CI = −0.52 to −0.26; *I^2^* = 19%, *p* = 0.27, *n* = 2) (Supplementary Figures 1C,D)]. The *α* diversity of gut microbiota showed a progressive decline with the progression of OSA severity (Supplementary Table 5).

**Figure 3:**
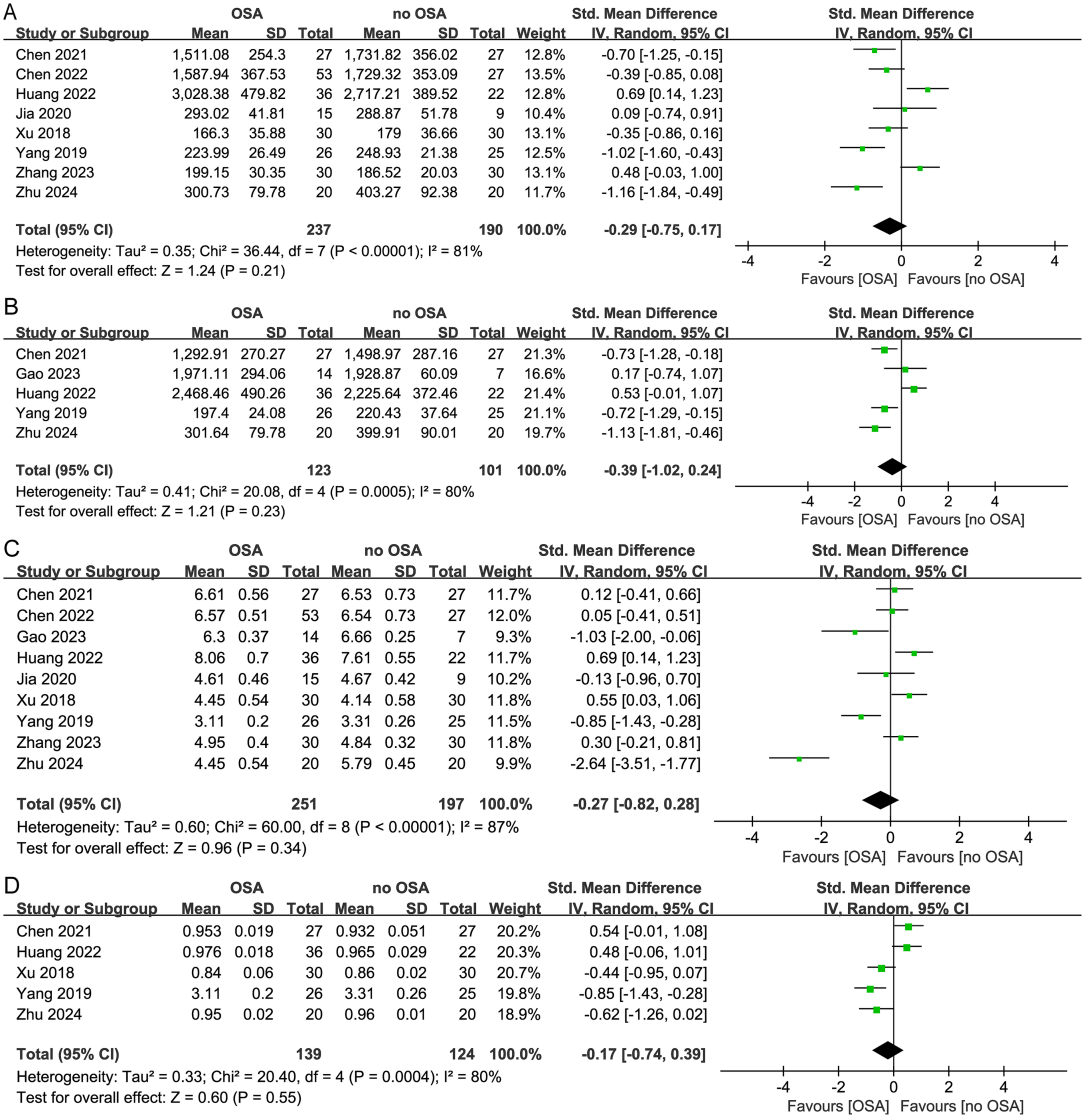
Forest plot of comparing oral microbiota alpha diversity in OSA and non-OSA populations. **(A)** Chao1 index; **(B)** Observed species; **(C)** Shannon index; **(D)** Simpson index.

Among the 10 studies focusing on oral microbiota, α diversity was reported in 9 of them. No significant difference was observed between groups with high heterogeneity across studies. The Chao1 index (SMD = −0.29, 95% CI = −0.75 to 0.17; *I^2^* = 81%, *p* < 0.00001, *n* = 8), Observed species (SMD = −0.39, 95% CI = −1.02 to 0.24; *I^2^* = 80%, *p* = 0.0005, *n* = 5), Shannon index (SMD = 0.27, 95% CI = −0.82 to 0.28; *I^2^* = 87%, *p* < 0.00001, *n* = 9), Simpson index (SMD = −0.17, 95% CI = −0.74 to 0.39; *I^2^* = 80%, *p* = 0.0004, *n* = 5) were not significantly different between OSA and non-OSA (Figures 3A–D), albeit those studies with higher heterogeneity. Sensitivity analyses excluding outlier studies maintained the significance of both indices, reinforcing the robustness of the conclusion. In subgroup analyses of the population, the adult OSA group exhibited lower *α* diversity compared to the control group [the Chao1 index (SMD = −0.56, 95% CI = −0.85 to −0.28 *I^2^* = 45%, *p* = 0.14, *n* = 4) (Supplementary Figure 2A)]. While no significant difference in α diversity was observed between the pediatric OSA group and non-OSA patients [the Chao1 index (SMD = −0.07, 95% CI = −0.83 to 0.70. *I^2^* = 87%, *p* < 0.0001, *n* = 4), Shannon index (SMD = −0.23; 95% CI = −1.37 to 0.92; *I^2^* = 93%, *p* < 0.00001, *n* = 4) (Supplementary Figures 2C,D)].

**Figure 4:**
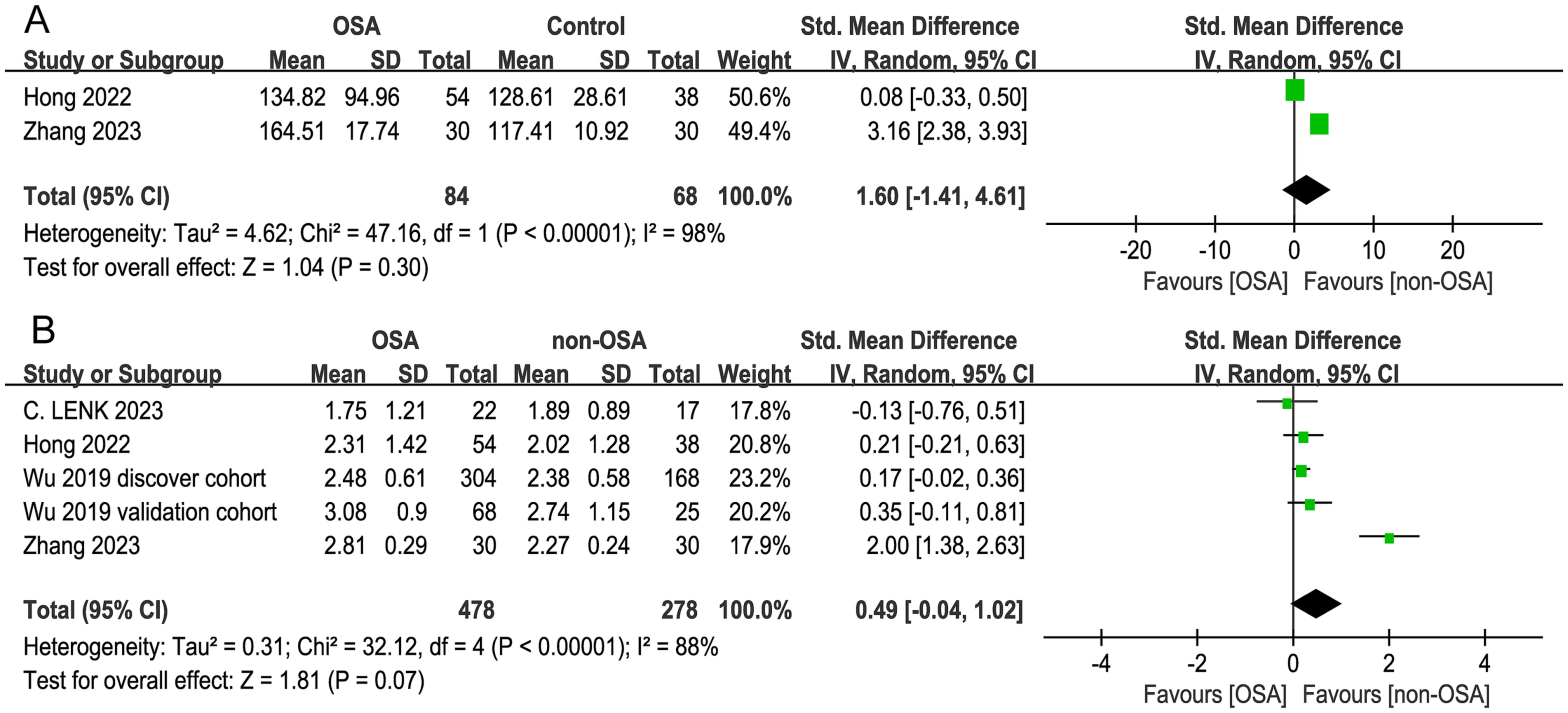
Forest plot of comparing respiratory tract microbiota alpha diversity in OSA and non-OSA populations. **(A)** Chao1 index; **(B)** Shannon index.

Among the 6 studies focusing on respiratory tract microbiota, α diversity was reported in 5 cohorts of them. No significant difference in α diversity was observed between groups with high heterogeneity across studies. The Chao1 index (SMD = 1.60, 95% CI = −1.41 to 4.61; *I^2^* = 98%, *p* < 0.00001, *n* = 2), Shannon index (SMD = 0.49, 95% CI = −0.04 to 1.02; *I^2^* = 88%, *p* < 0.00001, *n* = 5) were not significantly different between OSA and non-OSA (Figures 4A,B).

### 3.3 Beta diversity

Out of all the studies included, 17 studies reported *β*-diversity, predominantly utilizing UniFrac distances matrix and conducting PCoA analysis based on Bray–Curtis dissimilarity. Table 2 provides evidence supporting the findings: In terms of gut microbiota, 5 studies provide evidence supporting a discernible difference in the microbial composition between individuals with OSA and those non-OSA populations, while 2 studies suggest no significant distinction and 1 study suggest a significant distinction at the OUTs and genus level with no significant distinction at the phylum level. Regarding oral microbiota, 5 studies demonstrate a disparity in microbial structure between OSA patients and non-OSA populations, whereas 2 studies indicate no substantial variation. Concerning respiratory microbiota, 2 studies support the presence of dissimilarity in microbial composition between OSA patients and non-OSA populations, while another 2 studies suggest no notable difference.

**Table 2:** Summary of beta diversity assessments in the included studies.

Study	Sample	β diversity	Findings	Value
Wang et al. (2022)	Gut	PCoA	A significant difference in microbial composition between OSA and non-OSA	NR
Valentini et al. (2020)	Gut	PCoA based on unweighted UniFrac distances	NO significant difference in microbial composition between OSA and non-OSA	
		PCA (at the genus level)	A slight difference between OSA and non-OSA	*p* = 0.67
		NMDS based on Bray-Curtis distance	NO significant difference between OSA and non-OSA	*p* = 0.23
Wu et al. (2022)	Gut	PCoA (at the OTUs level)	A significant difference in gut microbial composition among OSA and non-OSA	*p* = 0.025
		PCoA (at the genus level)	A significant difference in gut microbial composition among OSA and non-OSA	*p* = 0.036
		PCoA (at the phylum level)	No significant difference in gut microbial composition among OSA and non-OSA	*p* = 0.203
Li et al. (2023)	Gut	PCoA	A significant difference in gut microbial composition among OSA and non-OSA	*p* = 0.044
Wang et al. (2021)	Gut	Unweighted UniFrac distances matrix	NO significant difference in gut microbial composition among OSA and non-OSA	*p* = 0.18
Zhang et al. (2022)	Gut	Unweighted UniFrac distances matrix	A significant difference in gut microbial composition among OSA and non-OSA	*p* = 0.001
Zhu et al. (2024)	Gut	PCoA	A significant difference in gut microbial composition among OSA and non-OSA	*p* < 0.05
Wang et al. (2024)	Gut	PCoA and NMDS	A significant difference in gut microbial composition among OSA and non-OSA	*p* < 0.05
Chen et al. (2022)	Oral	PCoA	A significant difference in gut microbial composition among OSA and non-OSA	*p* < 0.05
Chen et al. (2021)	Oral	Unweighted UniFrac distances matrix	A significant difference in gut microbial composition among OSA and non-OSA	*p* = 0.005
Jia et al. (2020)	Oral	Unweighted UniFrac distances matrix	No significant difference in gut microbial composition among OSA and non-OSA (at the OTU level)	*p* > 0.05
Zhang et al. (2023)	Oral	NMDS	A significant difference in microbial composition between the OSA and non-OSA	*p* = 0.001
		PcoA based on unweighted UniFrac distance	A significant difference in microbial composition between the OSA and non-OSA	*p* = 0.001
Huang et al. (2022)	Oral	PCoA	A significant difference in gut microbial composition among OSA and non-OSA	*p* = 0.004
Gao et al. (2023)	Oral	PCoA	NO significant difference in microbial composition between OSA and non-OSA	*p* > 0.05
Huang et al. (2022)	Oral	NR	A significant difference in gut microbial composition among OSA and non-OSA	NR
Lenk et al. (2023)	Respiratory tract	PCoA based on Bray-Curtis distance	No differences in microbial composition between the OSA and non-OSA	*p* = 0.9637
Zhang et al. (2023)	Respiratory tract	NMDS	A significant difference in microbial composition between the OSA and non-OSA	*p* = 0.001
		PcoA based on unweighted UniFrac distance	A significant difference in microbial composition between the OSA and non-OSA	*p* = 0.001
Wu et al. (2019)	Respiratory tract	PCoA based on weighted UniFrac distances	Significant differences between subjects with different severity of OSA non-OSA in the discovery cohort	*p* < 0.001
			Significant differences between subjects with different severity of OSA and non-OSA in the validation cohort	*p* = 0.04
Hong et al. (2022)	Respiratory tract	NMDS based on Bray-Curtis distance	No differences in microbial composition between the OSA and non-OSA	NR

OSA, Obstructive sleep apnea; non-OSA, non-obstructive sleep apnea populations; PCoA, principal coordinate analysis; PCoA, principal component analysis; NMDS, the nonmetric multi-dimensional scaling; NR, not reported.

### 3.4 Relative abundance of microbial taxa

**Figure 5:**
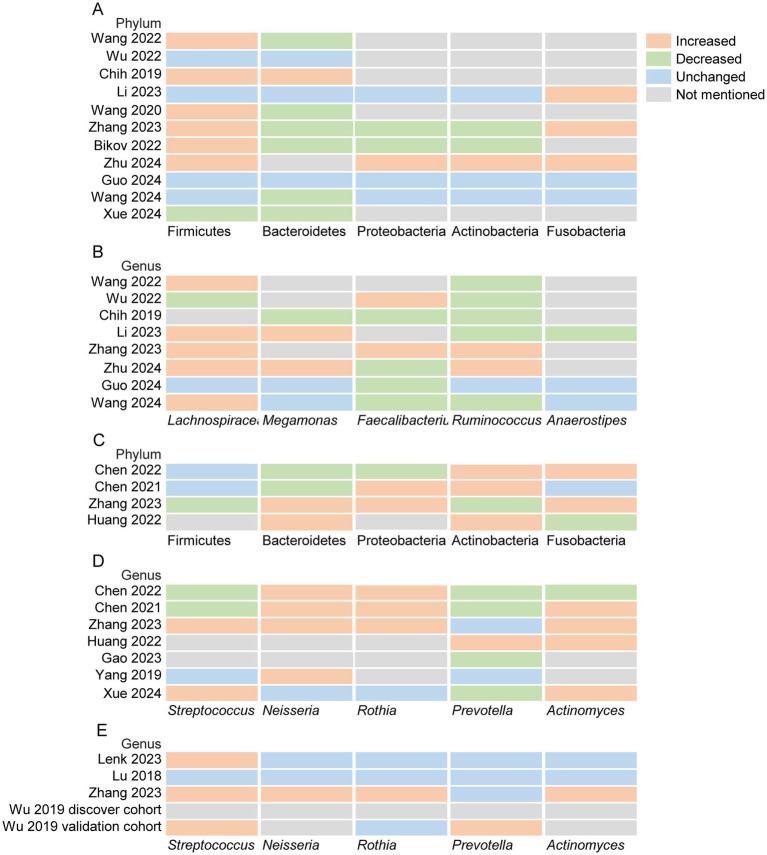
Comparison of relative abundance of microbiota in OSA patients and non-OSA population. **(A)** Heatmap analyses of gut microorganisms at the phylum level; **(B)** Heatmap analyses of gut microorganisms at the genus level; **(C)** Heatmap analyses of oral microorganisms at the phylum level; **(D)** Heatmap analyses of oral microorganisms at the genus level. **(E)** Heatmap analyses of respiratory tract microorganisms at the genus level.

Bacterial abundance data were available in 11 out of 13 studies investigating the gut microbiota, in 7 out of 10 studies examining the oral microbiota and in 4 out of 6 studies examining the respiratory tract microbiota, which facilitated the evaluation of alterations at phylum and genus levels relative to the abundance of gut and oral microbiota between individuals with OSA and those non-OSA populations. Figure 5 provide the comparison of relative abundance of bacteria in OSA patients and non-OSA population.

A Systematic analysis of 11 included studies revealed significant phylum-level alterations in the gut microbiota of patients with OSA compared to non-OSA populations. Notably, 54.5% (6/11) of studies demonstrated a statistically significant increase in the relative abundance of Firmicutes in OSA patients, while 66.7% (6/9) of studies reported a marked reduction in Bacteroidetes (Figure 5A). Critically, two pivotal studies (Valentini et al., 2020; Wang et al., 2024) corroborated these findings by identifying an elevated Firmicutes/Bacteroidetes (F/B) ratio in OSA cohorts, further validating the dysbiosis of these dominant bacterial phyla. At the genus level, integrative data from 8 studies highlighted distinct compositional shifts. A significant elevation in *Lachnospira* abundance was observed in 71.4% (5/7) of studies involving OSA patients. Conversely, 66.7% (4/6) of studies documented a decline in *Faecalibacterium*, and 62.5% (5/8) reported reduced levels of *Ruminococcus* (Figure 5B). These genus-specific perturbations may mechanistically link to the phylum-level imbalances, suggesting that OSA-associated metabolic or inflammatory pathways could selectively modulate microbial niches.

A synthesis of seven studies identified phylum-level perturbations in the oral microbiota of OSA patients compared to non-OSA controls. Among the phylum-level analyses, 75% (3/4) of studies reported a marked enrichment of Actinobacteria in OSA cohorts (Figure 5C). At the genus level, integrative data from five studies revealed consistent taxonomic shifts. A statistically significant elevation in abundance was observed for *Neisseria* (80%, 4/5 studies) and *Actinomyces* (80%, 4/5 studies), while *Rothia* demonstrated a 75% (3/4 studies) increase in OSA patients (Figure 5D). These genus-level alterations may synergistically contribute to OSA pathophysiology through multiple mechanisms.

Analysis of five studies investigating respiratory tract microbiota revealed genus-specific perturbations in OSA patients. At the phylum level, current evidence remains insufficient to establish consistent microbial structural changes. However, 75% (3/4) of studies demonstrated a significant elevation in *Streptococcus* abundance within OSA cohorts, suggesting a potential pathogenic role of this genus in upper airway inflammation or disease progression (Figure 5E).

Nine studies identified the bacterial markers distinguishing OSA from controls, including 6 gut and 3 oral. The bacterial markers were varied among different studies, including one bacterium and a model containing multiple bacteria. The AUCs were between 0.539–0.97, with a sensitivity between 37.2–76.7%, and specificity between 43.9–93.3% (Supplementary Table 6).

### 3.5 Sensitivity analysis and publication bias

The results of the heterogeneity assessment were consistent with our initial expectations. The forest plots of this meta-analysis revealed that the heterogeneity of Shannon index and Simpson index was consistently low in the study of gut microbiota. However, higher heterogeneity was observed for Chao1 index, Observed species, Shannon index and Simpson index in the study of respiratory tract microbiota of oral microbiota. Notably, among 27 studies analyzed here, Chao1 index and Shannon index emerged as the most frequently reported Alpha diversity indices. Sensitivity analyses were conducted along with Begg’s and Egger’s tests, for outcome indicators with sample sizes >5 and high heterogeneity. The test results indicated that the summary results of the sensitivity analysis remained stable for gut microbiota chao1 index (*p* = 0.764 in the Begg test and *p* = 0.809 in the Egger test) and gut microbiota Observed species index (*p* = 0.707 in the Begg test and *p* = 0.551 in the Egger test), oral microbiota chao1 index (*p* = 1.00 in the Begg test and *p* = 0.513 in the Egger test) and oral microbiota Shannon index (*p* = 0.917 in the Begg test and *p* = 0.914 in the Egger test). The outcomes revealed that all *p*-values associated with these indicators exceeded 0.05, indicating a lack of evidence supporting publication bias and providing additional support for the robustness of the conclusions derived from our meta-analysis.

## 4 Discussion

OSA can potentially induce dysbiosis in the gut microbiota through various mechanisms. Firstly, the composition of the microbiota is affected by oxygen partial pressure (Albenberg et al., 2014), and intermittent hypoxemia associated with OSA may lead to alterations in the gut microbiota (Zhang et al., 2021). Secondly, sleep fragmentation resulting from OSA also plays a significant role in gut dysbiosis. Smith et al. (2019) discovered that gut microbes could influence sleep quality in humans through the brain-gut-microbiota axis (BGMA). Dysbiosis of the gut microbiota can potentially impact neurotransmitter production, thereby contributing to sleep disorders (Neroni et al., 2021). Furthermore, in patients with OSA, a diet rich in high-fat and high-carbohydrate content have been shown to influence microbial composition (Yatsunenko et al., 2012).

Our study represents a pioneering meta-analysis that comprehensively synthesizes the gut, respiratory tract and oral microbiota of individuals with OSA. We conducted a meta-analysis encompassing 27 studies to investigate alterations in microbiota diversity and microbiota abundance at the phylum and genus levels across various regions of the gastrointestinal oral and respiratory tract in a cohort of 2073 participants (1,381 patients with OSA and 692 controls), including 1804 adults and 321 children. We assessed the microbiota using measures such as microbial group abundance, alpha diversity, taxonomic composition alterations, and beta diversity analysis to evaluate shifts in microbiota community composition. The collected evidence demonstrated that the diversity of the microbiota of OSA patients decreased, as well h as an increase in Firmicutes and a decrease in Bacteroidetes in the intestines of OSA patients. These consistent trends provide clues for further exploring the pathogenesis of OSA and may be of reference value for the future development of OSA-related microbiota diagnostic markers or intervention targets.

The stability of microbial ecosystems is influenced by the diversity of microorganisms present. Alpha diversity serves as a widely used indicator for assessing microbial ecological dysbiosis, reflecting the relative abundance of microbial species within a given community across spatial and temporal scales. The alpha diversity indices encompass Chao1 index, ACE, Simpson index and Shannon index, each emphasizing distinct microbial characteristics. The Chao1 index, and ACE index are indicative of community richness, while the Shannon index and Simpson index reflect microbiota community homogeneity (Kim et al., 2017). The distinction between Shannon’s and Simpson’s indices lies in the fact that Simpson’s index places greater emphasis on the relative abundance among different species, whereas Shannon’s index primarily focuses on species richness (Kim et al., 2017). Gut microbiota diversity serves as a crucial health indicator, and reduced *α*-diversity may be deemed detrimental to the host due to the proliferation of pathogenic microorganisms (Le Chatelier et al., 2013). The meta-analysis revealed a significant decrease in the Chao 1 index, and Shannon index of gut microbiota among patients with OSA. Despite the heterogeneity observed across studies in terms of geographical region, ethnic background, and research methodology, the statistically significant disparity in α-diversity highlights that patients with OSA exhibit gut ecological dysregulation characterized by diminished phylogenetic abundance and disruption of microbiota homogeneity. This novel finding offers a fresh perspective for investigating the potential etiology of OSA. However, no statistically significant differences were observed in alpha diversity within the oral cavity and respiratory tracts of patients with OSA. This may be limited by the variation of sample types and sites and the insufficient number of included studies. Given the limited number of available studies, it is imperative to exercise caution when interpreting these findings. Beta diversity is influenced by variations in species composition across multiple samples. Our aggregated Beta diversity findings provide evidence for dissimilarities in microbiota structure between individuals with OSA and those non-OSA population.

The gut microbiota represents a complex and diverse ecosystem, wherein the collected evidence alterations in the compositional profile of gut microbiota in individuals with OSA. Enrichment of Firmicutes at the phylum level was observed, along with an increased abundance of *Lachnospira* at the genus level, while a decreased relative abundance of *Ruminococcaceae* was noted. The gut microbiota profiles associated with OSA may exhibit intra-genus species variation, which surpasses the resolution of 16S sequencing and necessitates whole-genome metagenomic sequencing for further evaluation and identification of disease-specific biomarkers. The composition of gut microbiota may exhibit interpopulation variations, encompassing factors such as race, age, gender, obesity status, and severity of OSA (Lang and Schnabl, 2020). The collected evidence revealed distinct characteristics in both adult and pediatric OSA patients, the two populations analyzed in the subgroup analyses also yielded differing conclusions. Studies have demonstrated that pediatric OSA patients exhibit a greater number of metabolites associated with abnormal carbohydrate and amino acid metabolism compared to adult OSA patients, potentially attributable to distinct pathogenic mechanisms (Xu et al., 2018). Additionally, adult OSA appears to be more strongly linked to obesity, as evidenced by the characteristics of the studies reviewed. The acquisition of adequate relative abundance data for validation necessitates further investigations in the future.

The collected evidence demonstrated that the gut tracts of patients with OSA exhibited an increased relative abundance of Firmicutes and *Lachnospira* compared to the non-OSA population. The dynamic equilibrium between the obligate anaerobic bacteria Firmicutes and Bacteroidetes is a defining characteristic of the human gut microbiota (Human Microbiome Project Consortium, 2012). The increased abundance of Firmicutes may suggest dysregulation in the physiological interactions between the host and gut microbiota in patients with OSA (Moreno-Indias et al., 2015; Moreno-Indias et al., 2016). It has been reported that an increased relative abundance of Firmicutes is accompanied by elevated levels of endotoxin in the bloodstream, thereby triggering a systemic inflammatory response (Poppleton et al., 2017). The *Bacteroides* plays a crucial role in carbohydrate and fiber fermentation, producing short-chain fatty acids (SCFAs) such as butyrate, acetate, and propionate. SCFAs are essential in maintaining human health by providing the primary source of nutrition and energy for colon cells, protecting the intestinal mucosal barrier, reducing inflammation in the host, and enhancing intestinal peristalsis (Li and Shi, 2023). The abundance of *Lachnospira* exhibited a positive correlation with TMAO levels (Zhu et al., 2016). TMAO has been implicated in the regulation of cholesterol and sterol metabolism, as well as the promotion of atherosclerosis, platelet aggregation, and cardiovascular events (Zhu et al., 2016; Randrianarisoa et al., 2016). In addition, our results also found that there was a tendency for a decrease in the relative abundance of *Ruminococcus*. R*uminococcus* not only synthesizes (SCFA), but also actively participates in the metabolism of bile acids (Peters et al., 2022). *Ruminococcus* possesses 7 *α*-dehydroxylation and 7 *β*-dehydrogenation genes that facilitate the biotransformation of bile acids (Vital et al., 2019; Ikegami and Honda, 2018). Dysfunction in the secretion and reabsorption of bile acids may constitute a significant characteristic associated with insulin resistance, obesity, and type 2 diabetes (Yang et al., 2018; Kuipers et al., 2014). Furthermore, the Farnesoid X receptor and G protein-coupled bile acid receptor 1 (Gpbar1) exert pivotal roles in governing glucose, lipid, and energy metabolism regulation (Kuipers et al., 2014). Although metabolic disorders such as hyperinsulinemia, insulin resistance, and obesity commonly coexist with OSA (Carneiro and Zanella, 2018). Chronic intermittent hypoxia (CIH) is a key characteristic of OSA. Animal experiments demonstrated that CIH induction altered the diversity and composition of gut microbiota, specifically reducing beneficial bacteria while increasing harmful bacteria/opportunistic pathogens, and increased typical pro-inflammatory mediators in serum including CRP, TNF-α, IL-6, IL-8 and NF-κB. In addition, microbiota related metabolic pathways, including cAMP signaling pathway, phenylalanine metabolism, prolactin signaling pathway, et al. were significantly affected. These suggest that dysbiosis of gut microbiome was associated with systemic inflammation and metabolism disorder, and emerges as a mediator for CIH and its consequences (Li and Shi, 2023; Zhang et al., 2022). Furthermore, the α diversity of gut microbiota showed a progressive decline with the progression of OSA severity, further proved the causal relationships between microbiota and OSA.

Furthermore, the collected evidence also observed an increase in the relative abundance of Actinobacteria, *Neisseria*, *Rothia*, and *Actinomyce*s within the oral microbiota. A similar pattern was observed in the respiratory microbiota: although analysis of the collected evidence revealed no significant differences in the alpha diversity of the respiratory microbiota among patients with obstructive sleep apnea (OSA), four of the five Shannon indices evaluated in this study demonstrated statistically significant elevations, contrary to the results observed in the gut microbiota. These microbial alterations may potentially contribute to the pathogenesis of multisystemic and multiorgan disorders associated with OSA. However, given the limited number of included studies and the heterogeneity observed among them, further empirical studies are required to delineate the underlying mechanisms driving this phenomenon.

The present study has certain limitations. Firstly, a limited number of studies meeting the inclusion criteria failed to conduct subgroup analysis on changes in microbiota based on sample characteristics, thereby impeding the identification of potential confounding factors. Furthermore, there is a scarcity of literature documenting both domestic and international clinical investigations elucidating the regulatory role of OSA on host metabolism through bacterial microbiota. In future research, longitudinal metabolomics and multi-omics studies should be further employed to elucidate the microbial ligands and metabolites that interact with host immunity in multi-center and large-scale clinical trials. Additionally, the gut microbial profiles associated with OSA may exhibit intra-genus species variation, which surpasses the resolution of 16S sequencing and necessitates whole-genome metagenomic sequencing for further evaluation and identification of disease-specific biomarkers. Moreover, significant methodological variations were observed in the demographic characteristics of the included studies, particularly regarding the sampling techniques employed for oral cavity specimens as well as storage protocols utilized for fecal samples. We advocate for standardized methodology utilization in microbiota analyses while emphasizing the necessity for larger-scale studies encompassing comprehensive data on participants’ dietary habits and sleep parameters to substantiate our findings. Furthermore, given the paucity of standardized microbiome datasets and substantial methodological heterogeneity across studies, the current analytical framework precludes definitive conclusions regarding dysbiotic patterns in OSA-associated respiratory microbiota, it is imperative for future investigations to incorporate comprehensive microbiota data including bacteria, viruses, and fungi to enhance scholarly rigor and scientific nature.

## 5 Conclusion

Our meta-analysis comprehensively summarizes the alterations in microbiota richness and diversity among patients with OSA, as well as variations in the composition of disease-specific microorganisms. The majority of published studies support the hypothesis that patients with OSA exhibit altered microbiota diversity, particularly a reduction in the alpha diversity of their gut microbiota. The findings further highlight the impact of microbiota on OSA, emphasizing the pro-inflammatory environment that arises due to intricate interactions between microbiota and the host. Furthermore, changes in the relative abundance of microbial communities in difference cavities of body (gut, oral, respiratory tract) may occur, based on variations in the age of patients. This study serves as a fundamental reference for future investigations into potential pathogenic mechanisms and therapeutic strategies targeting OSA.

## Data Availability

The original contributions presented in the study are included in the article/Supplementary material, further inquiries can be directed to the corresponding authors.
