# Supplementary material for: Microbial dysbiosis in obstructive sleep apnea: a systematic review and meta-analysis
Source: Front Microbiol. 2025 May 15;16:1572637. doi: 10.3389/fmicb.2025.1572637 (PMC12119640; doi:10.3389/fmicb.2025.1572637)
Supplement: Supplementary file 1 [file Data_Sheet_1.DOCX]

Supplementary Material

**Supplementary Table 1.** Systematic search detail.

**Supplementary Table 2.** Alpha diversity index of gut microbiota between OSA and non-OSA.

**Supplementary Table 3.** Alpha diversity index of oral microbiota between OSA and non-OSA.

**Supplementary Table 4.** Alpha diversity index of respiratory tract microbiota between OSA and non-OSA.

**Supplementary Table 5.** Alpha diversity of the different severity of OSA.

**Supplementary Table 6.** Receiver operating characteristic analysis of microbiota markers of the distinguishing OSA from controls included studies.

**Supplementary Figure 1:** Forest plot comparing alpha diversity of the gut microbiota in adult and pediatric OSA and non-OSA populations.

**Supplementary Figure 2:** Forest plot comparing alpha diversity of the oral microbiota in adult and pediatric OSA and non-OSA populations.

**Supplementary Figure 3:** Sensitivity analysis of gut and oral microbiota. A-D: Sensitivity analysis of gut microbiota.

**Supplementary Figure 4:** Funnel plot of gut and oral microbiota. A-D: Funnel plot of gut microbiota.

**Supplementary Table 1. Systematic search detail.**

| Database | Search strategy |
| --- | --- |
| PubMed | ((((((((((((((((((((((("Microbiota"[Mesh]) OR (Microbiota[Title/Abstract])) OR (Microbiotas[Title/Abstract])) OR (Microbial Community[Title/Abstract])) OR (Community, Microbial[Title/Abstract])) OR (Microbial Communities[Title/Abstract])) OR (Microbial Community Composition[Title/Abstract])) OR (Community Composition, Microbial[Title/Abstract])) OR (Composition, Microbial Community[Title/Abstract])) OR (Microbial Community Compositions[Title/Abstract])) OR (Microbial Community Structure[Title/Abstract])) OR (Community Structure, Microbial[Title/Abstract])) OR (Microbial Community Structures[Title/Abstract])) OR (Microbiome[Title/Abstract])) OR (Microbiomes[Title/Abstract])) OR (Human Microbiome[Title/Abstract])) OR (Human Microbiomes[Title/Abstract])) OR (Microbiome, Human[Title/Abstract])) OR (microbiota[Title/Abstract])) OR (micromicrobiota[Title/Abstract])) OR (dysbiosis[Title/Abstract])) OR (bacteria[Title/Abstract])) OR (ecosystem[Title/Abstract])) AND ((((((((((((((("Sleep Apnea, Obstructive"[Mesh]) OR (Sleep Apnea, Obstructive[Title/Abstract])) OR (Apneas, Obstructive Sleep[Title/Abstract])) OR (Obstructive Sleep Apneas[Title/Abstract])) OR (Sleep Apneas, Obstructive[Title/Abstract])) OR (Obstructive Sleep Apnea Syndrome[Title/Abstract])) OR (Obstructive Sleep Apnea[Title/Abstract])) OR (OSAHS[Title/Abstract])) OR (Syndrome, Sleep Apnea, Obstructive[Title/Abstract])) OR (Sleep Apnea Syndrome, Obstructive[Title/Abstract])) OR (Apnea, Obstructive Sleep[Title/Abstract])) OR (Sleep Apnea Hypopnea Syndrome[Title/Abstract])) OR (Syndrome, Obstructive Sleep Apnea[Title/Abstract])) OR (Upper Airway Resistance Sleep Apnea Syndrome[Title/Abstract])) OR (Syndrome, Upper Airway Resistance, Sleep Apnea[Title/Abstract])) |
| The Cochrane Library | (Sleep Apnea, Obstructive):ti,ab,kw OR(Apneas, Obstructive Sleep):ti,ab,kw OR(Obstructive Sleep Apneas):ti,ab,kw OR(Sleep Apneas, Obstructive):ti,ab,kw OR(Obstructive Sleep Apnea Syndrome):ti,ab,kw OR(Obstructive Sleep Apnea):ti,ab,kw OR(OSAHS):ti,ab,kw OR(Syndrome, Sleep Apnea, Obstructive):ti,ab,kw OR(Sleep Apnea Syndrome, Obstructive):ti,ab,kw OR(Apnea, Obstructive Sleep):ti,ab,kw OR(Sleep Apnea Hypopnea Syndrome):ti,ab,kw OR(Syndrome, Obstructive Sleep Apnea):ti,ab,kw OR(Upper Airway Resistance Sleep Apnea Syndrome):ti,ab,kw OR(Syndrome, Upper Airway Resistance, Sleep Apnea):ti,ab,kw OR(Obstructive Sleep Apneas):ti,ab,kw OR(Obstructive Sleep Apnea Syndrome):ti,ab,kw OR(Obstructive Sleep Apnea):ti,ab,kw OR(OSAHS):ti,ab,kw OR(Sleep Apnea Hypopnea Syndrome):ti,ab,kw OR(Syndrome, Obstructive Sleep Apnea):ti,ab,kw OR(Upper Airway Resistance Sleep Apnea Syndrome):ti,ab,kw OR(Syndrome, Upper Airway Resistance, Sleep Apnea):ti,ab,kw AND (Microbiota):ti,ab,kw OR(Microbiotas):ti,ab,kw OR(Microbial Community):ti,ab,kw OR(Community, Microbial):ti,ab,kw OR(Microbial Communities):ti,ab,kw OR(Microbial Community Composition):ti,ab,kw OR(Community Composition, Microbial):ti,ab,kw OR(Composition, Microbial Community):ti,ab,kw OR(Microbial Community Compositions):ti,ab,kw OR(Microbial Community Structure):ti,ab,kw OR(Community Structure, Microbial):ti,ab,kw OR(Microbial Community Structures):ti,ab,kw OR(Microbiome):ti,ab,kw OR(Microbiomes):ti,ab,kw OR(Human Microbiome):ti,ab,kw OR(Human Microbiomes):ti,ab,kw OR(Microbiome, Human):ti,ab,kw OR(flora):ti,ab,kw OR(microflora):ti,ab,kw OR(dysbiosis):ti,ab,kw OR(bacteria):ti,ab,kw OR(ecosystem):ti,ab,kw OR(Eubacteria):ti,ab,kw |
| Web of Science | (TS=(Microbiota) OR AB=(Microbiota OR Microbiotas OR Microbial Community OR Community, Microbial OR Microbial Communities OR Microbial Community Composition OR Community Composition, Microbial OR Composition, Microbial Community OR Microbial Community Compositions OR Microbial Community Structure OR Community Structure, Microbial OR Microbial Community Structures OR Microbiome OR Microbiomes OR Human Microbiome OR Human Microbiomes OR Microbiome, Human OR microbiota OR micromicrobiota OR dysbiosis OR bacteria OR ecosystem)) AND (TS=(Sleep Apnea, Obstructive) OR AB=(Sleep Apnea, Obstructive OR Apneas, Obstructive Sleep OR Obstructive Sleep Apneas OR Sleep Apneas, Obstructive OR Obstructive Sleep Apnea Syndrome OR Obstructive Sleep Apnea OR OSAHS OR Syndrome, Sleep Apnea, Obstructive OR Sleep Apnea Syndrome, Obstructive OR Apnea, Obstructive Sleep OR Sleep Apnea Hypopnea Syndrome OR Syndrome, Obstructive Sleep Apnea OR Upper Airway Resistance Sleep Apnea Syndrome OR Syndrome, Upper Airway Resistance, Sleep Apnea)) |
| Embase | ‘Microbiota’:ab,ti OR ‘Microbiotas’:ab,ti OR ‘Microbial Community’:ab,ti OR ‘Community, Microbial’:ab,ti OR ‘Microbial Communities’:ab,ti OR ‘Microbial Community Composition’:ab,ti OR ‘Community Composition, Microbial’:ab,ti OR ‘Composition, Microbial Community’:ab,ti OR ‘Microbial Community Compositions’:ab,ti OR ‘Microbial Community Structure’:ab,ti OR ‘Community Structure, Microbial’:ab,ti OR ‘Microbial Community Structures’:ab,ti OR ‘Microbiome’:ab,ti OR ‘Microbiomes’:ab,ti OR ‘Human Microbiome’:ab,ti OR ‘Human Microbiomes’:ab,ti OR ‘Microbiome, Human’:ab,ti OR ‘microbiota’:ab,ti OR ‘micromicrobiota’:ab,ti OR ‘dysbiosis’:ab,ti OR ‘bacteria’:ab,ti OR ‘ecosystem’:ab,ti OR ‘Eubacteria':ab,ti  ＆  ‘Sleep Apnea, Obstructive’:ab,ti OR ‘Apneas, Obstructive Sleep’:ab,ti OR ‘Obstructive Sleep Apneas’:ab,ti OR ‘Sleep Apneas, Obstructive’:ab,ti OR ‘Obstructive Sleep Apnea Syndrome’:ab,ti OR ‘Obstructive Sleep Apnea’:ab,ti OR ‘OSAHS’:ab,ti OR ‘Syndrome, Sleep Apnea, Obstructive’:ab,ti OR ‘Sleep Apnea Syndrome, Obstructive’:ab,ti OR ‘Apnea, Obstructive Sleep’:ab,ti OR ‘Sleep Apnea Hypopnea Syndrome’:ab,ti OR ‘Syndrome, Obstructive Sleep Apnea’:ab,ti OR ‘Upper Airway Resistance Sleep Apnea Syndrome’:ab,ti OR ‘Syndrome, Upper Airway Resistance, Sleep Apnea':ab,ti OR ‘Obstructive Sleep Apneas’:ab,ti OR ‘Obstructive Sleep Apnea Syndrome’:ab,ti OR ‘Obstructive Sleep Apnea’:ab,ti OR ‘OSAHS’:ab,ti OR ‘Sleep Apnea Hypopnea Syndrome’:ab,ti OR ‘Syndrome, Obstructive Sleep Apnea’:ab,ti OR ‘Upper Airway Resistance Sleep Apnea Syndrome’:ab,ti OR ‘Syndrome, Upper Airway Resistance, Sleep Apnea':ab,ti |

**Supplementary Table 2.** Alpha-diversity index of gut microbiota between OSA and non-OSA.

| Study | Site | Chao1 index | |  | Observed species | |  | Shannon index | |  | Simpson index | |
| --- | --- | --- | --- | --- | --- | --- | --- | --- | --- | --- | --- | --- |
|  |  | OSA | non-OSA |  | OSA | non-OSA |  | OSA | non-OSA |  | OSA | non-OSA |
| Valentini 2020[1] | stool | 213.60±11.20 | 244.10±21.90 |  | 214.30±11.4 | 245.10±21.90 |  | 2.22±0.30 | 2.11±0.20 |  |  |  |
| Wang 2021[2] | stool | 1159.38±588.91 | 1192.77±465.53 |  |  |  |  | 4.84±2.03 | 5.47±1.87 |  | 0.081±0.039 | 0.067±0.026 |
| Wang 2022[3] | stool | 203.89±67.32 | 238.89±81.86 |  | 126.20±67.20 | 169.50±67.20 |  | 3.2±0.46 | 3.42±0.27 |  |  |  |
| Wu 2022[4] | stool | 336.03±47.84 | 349.50±32.61 |  | 262.40±24.10 | 275.20±31.50 |  | 2.91±0.39 | 3.00±0.43 |  |  |  |
| Zhang 2023[5] | stool | 212.18±67.88 | 288.92±172.95 |  |  |  |  | 4.63±0.75 | 5.02±1.22 |  |  |  |
| Guo 2024[6] | stool | 251.53±69.45 | 306.38±73.72 |  | 238.99±67.05 | 299.46±47.87 |  | 2.78±0.63 | 3.10±0.29 |  | 0.143±0.102 | 0.122±0.084 |
| Liu 2024[7] | stool |  |  |  |  |  |  | 3.22±1.55 | 2.92±0.83 |  |  |  |
| Wang 2024[8] | stool | 1666.65±607.98 | 1474.49±533.93 |  | 1261.01±494.23 | 1302.08±433.31 |  | 4.93±1.42 | 5.50±1.37 |  | 0.954±0.025 | 0.967±0.176 |
| Xue 2024[9] | stool |  |  |  | 4906.79±729.52 | 4712.92±514.88 |  | 6.17±0.93 | 5.84±1.07 |  |  |  |

**Supplementary Table 3.** Alpha-diversity index of oral microbiota between OSA and non-OSA.

| Study | Site | Chao1 index | |  | Observed species | |  | Shannon index | |  | Simpson index | |
| --- | --- | --- | --- | --- | --- | --- | --- | --- | --- | --- | --- | --- |
|  |  | OSA | non- OSA |  | OSA | non- OSA |  | OSA | non- OSA |  | OSA | non- OSA |
| Chen 2022 [10] | Saliva | 1587.94±367.50 | 1729.32±353.09 |  |  |  |  | 6.57±0.51 | 6.54±0.73 |  |  |  |
| Chen 2021 [11] | Saliva | 1511.08±254.30 | 1731.82±356.02 |  | 1292.91±270.30 | 1498.97±287.2 |  | 6.61±0.56 | 6.53±0.73 |  | 0.95±0.02 | 0.93±0.05 |
| Jia 2020 [12] | Saliva | 293.02±41.81 | 288.87±51.78 |  |  |  |  | 4.61±0.46 | 4.67±0.42 |  |  |  |
| Huang 2022[13] | Saliva | 3028.38±479.8 | 2717.21±389.52 |  | 2468.46±490.30 | 2225.64±372.5 |  | 8.06±0.70 | 7.61±0.55 |  | 0.98±0.02 | 0.97±0.029 |
| Gao 2023[14] | Oral rinse |  |  |  | 1971.11±294.10 | 1928.87±60.09 |  | 6.30±0.37 | 6.66±0.25 |  |  |  |
| Xu 2018[15] | Oral swab | 166.30±35.88 | 179.00±36.66 |  |  |  |  | 4.45±0.54 | 4.14±0.58 |  | 0.84±0.06 | 0.86±0.02 |
| Yang 2019[16] | Oral swab | 223.99±26.49 | 248.93±21.38 |  | 197.40±24.08 | 220.43±37.64 |  | 3.11±0.20 | 3.31±0.26 |  | 3.11±0.20 | 3.31±0.26 |
| Zhang 2023[17] | Oral swab | 199.15±30.35 | 186.52±20.03 |  |  |  |  | 4.95±0.40 | 4.84±0.32 |  |  |  |
| Zhu 2024[18] | Oral swab | 300.73±79.78 | 403.27±92.38 |  | 301.64±79.78 | 399.91±90.01 |  | 4.45±0.54 | 5.79±0.45 |  | 0.95±0.02 | 0.96±0.01 |

**Supplementary Table 4.** Alpha-diversity index of respiratory tract microbiota between OSA and non-OSA.

| Study | Site | Chao1 index | |  | Ace index | |  | Shannon index | |  | Simpson index | |
| --- | --- | --- | --- | --- | --- | --- | --- | --- | --- | --- | --- | --- |
|  |  | OSA | non-OSA |  |  |  |  | OSA | non-OSA |  | OSA | non-OSA |
| LENK 2023[19] | Nasal swab |  |  |  |  |  |  | 1.75±1.21 | 1.81±0.68 |  |  |  |
| Hong 2022[20] | Nasal swab | 134.82±94.96 | 128.61±28.61 |  |  |  |  | 2.31±1.42 | 2.02±1.28 |  | 0.6435 ± 0.2827 | 0.6095 ± 0.2683 |
| Wu 2019 discover cohort[21] | Nasal lavage |  |  |  |  |  |  | 2.48±0.61 | 2.38±0.58 |  |  |  |
| Wu 2019 validation cohort[21] | Nasal lavage |  |  |  |  |  |  | 3.08±0.90 | 2.74±1.15 |  |  |  |
| Zhang 2023[17] | Nasal swab | 164.51±17.74 | 117.41±10.92 |  |  |  |  | 2.81±0.29 | 2.27±0.24 |  |  |  |

**Supplementary Table** **5.** Alpha diversity of the different severity of OSA.

| Indictors | Study Year | Sample Type | Mild OSA | Moderate OSA | Severe OSA |
| --- | --- | --- | --- | --- | --- |
| Chao1 index | Guo 2024[6] | Stool | 273.54±83.58 | 238.17±231.31 | 237.65±89.21 |
|  | Wang 2021[2] | Stool | 1236.40±595.02 | 1254.17±231.31 | 1061.67±551.46 |
|  | Wang 2024[8] | Stool | 1765.82±447.68 | 1654.53±538.27 | 1204.51±603.34 |
| Shannon index | Guo 2024[6] | Stool | 2.87±0.56 | 2.66±0.96 | 2.77±0.77 |
|  | Wang 2021[2] | Stool | 6.36±1.04 | 6.43±0.89 | 5.69±1.18 |
|  | Wang 2024[8] | Stool | 6.96±0.55 | 6.75±0.86 | 5.95±1.05 |
| Simpson index | Guo 2024[6] | Stool | 0.128±0.084 | 0.153±0.110 | 0.154±0.123 |
|  | Wang 2024[8] | Stool | 0.968±0.011 | 0.956±0.019 | 0.940±0.029 |
| Shannon index | Wu 2019 discover cohort [21] | Nasal lavage | 2.31±0.33 | 2.46±0.47 | 2.67±0.68 |
|  | Wu 2019 validation cohort[21] | Nasal lavage | 2.85±1.11 | 2.98±0.94 | 3.27±0.70 |

**Supplementary Table 6** Receiver operating characteristic analysis of microbiota markers of the distinguishing OSA from controls included studies.

| Study Year | Sample Type | Marker | AUC (95%CI) | Sensitivity (%) | Specificity (%) |
| --- | --- | --- | --- | --- | --- |
| Wu 2024 [4] | Stool | *Faecalibacterium* | 0.689 (0.572-0.808) | 76.7 | 66.7 |
| Wu 2024[4] | Stool | unclassified *Ruminococcaceae* | 0.641 (0.525-0.756) | 76.7 | 48.9 |
| Wu 2024[4] | Stool | *Roseburia* | 0.539 (0.412-0.665) | 37.2 | 91.1 |
| Zhu 2024[22] | Stool | *Pseudomonas* | 0.798 (0.674-0.921) | NR | NR |
| Zhu 2024[22] | Stool | *Megamonas* | 0.678 (0.537-0.818) | NR | NR |
| Ko 2019[23] | Stool | 29 genera | 0.789 (NR) | NR | NR |
| Chen 2021[11] | Saliva | 5 generas inclouding (*Cronobacter, Escherichia, Kingella, Oribacterium,* and *Peptostreptococcus*) | 0.919 (85.35-98.51) | NR | NR |
| Huang 2022 [20] | Saliva | 10 genera inclouding (*Prevotella, Parvimonas, Bacteroidetes, Bacteroides, Klesbsiella, Bifidobacterium, Capnocytophaga, Escherichia, Lactobacillus,Lautropia*) | 0.97 (NR) | NR | NR |
| Ko 2019[24] | Oral swab | *Porphyromonas and Aggregatibacter* | 0.759 (NR) | NR | NR |


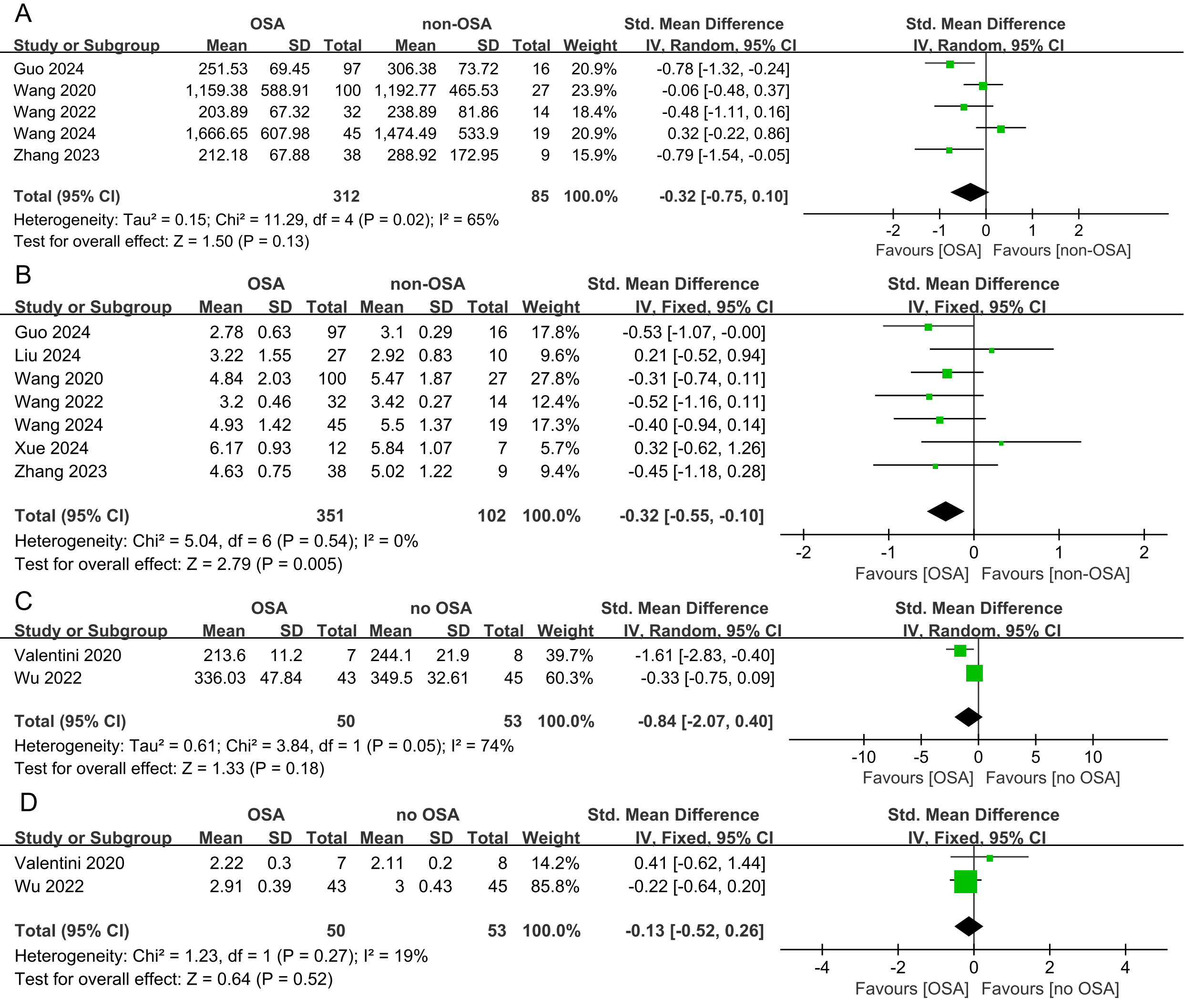


**Supplementary Figure 1:** Forest plot comparing alpha diversity of the gut microbiota in adult and pediatric OSA and non-OSA populations. A: Adult Chao1 index; B: Adult Shannon index; C: Pediatric Chao1 index; D: Pediatric Shannon index.


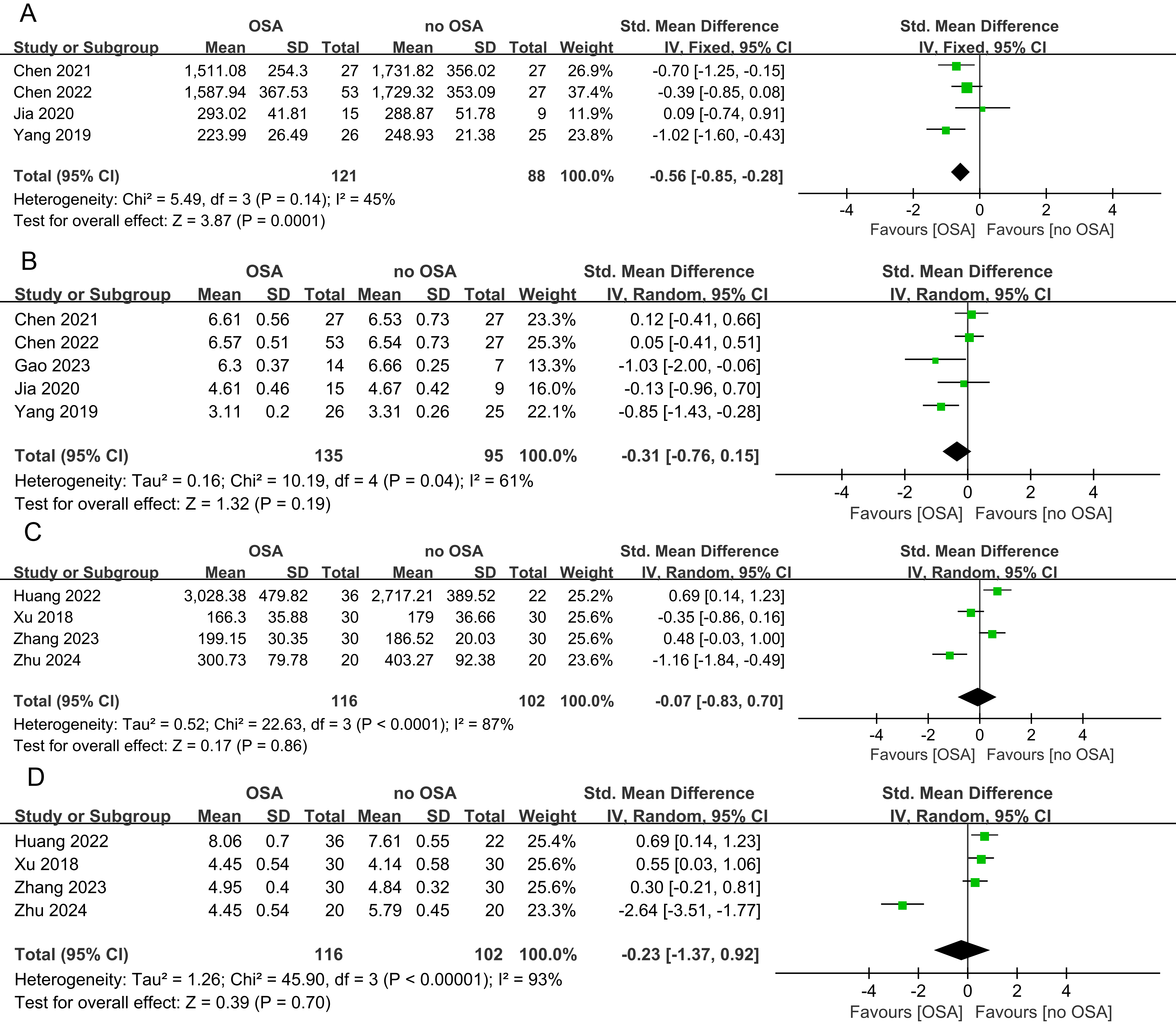


**Supplementary Figure 2:** Forest plot comparing alpha diversity of the oral microbiota in adult and pediatric OSA and non-OSA populations. A: Adult Chao1 index; B: Adult Shannon index; C: Pediatric Chao1 index; D: Pediatric Shannon index.


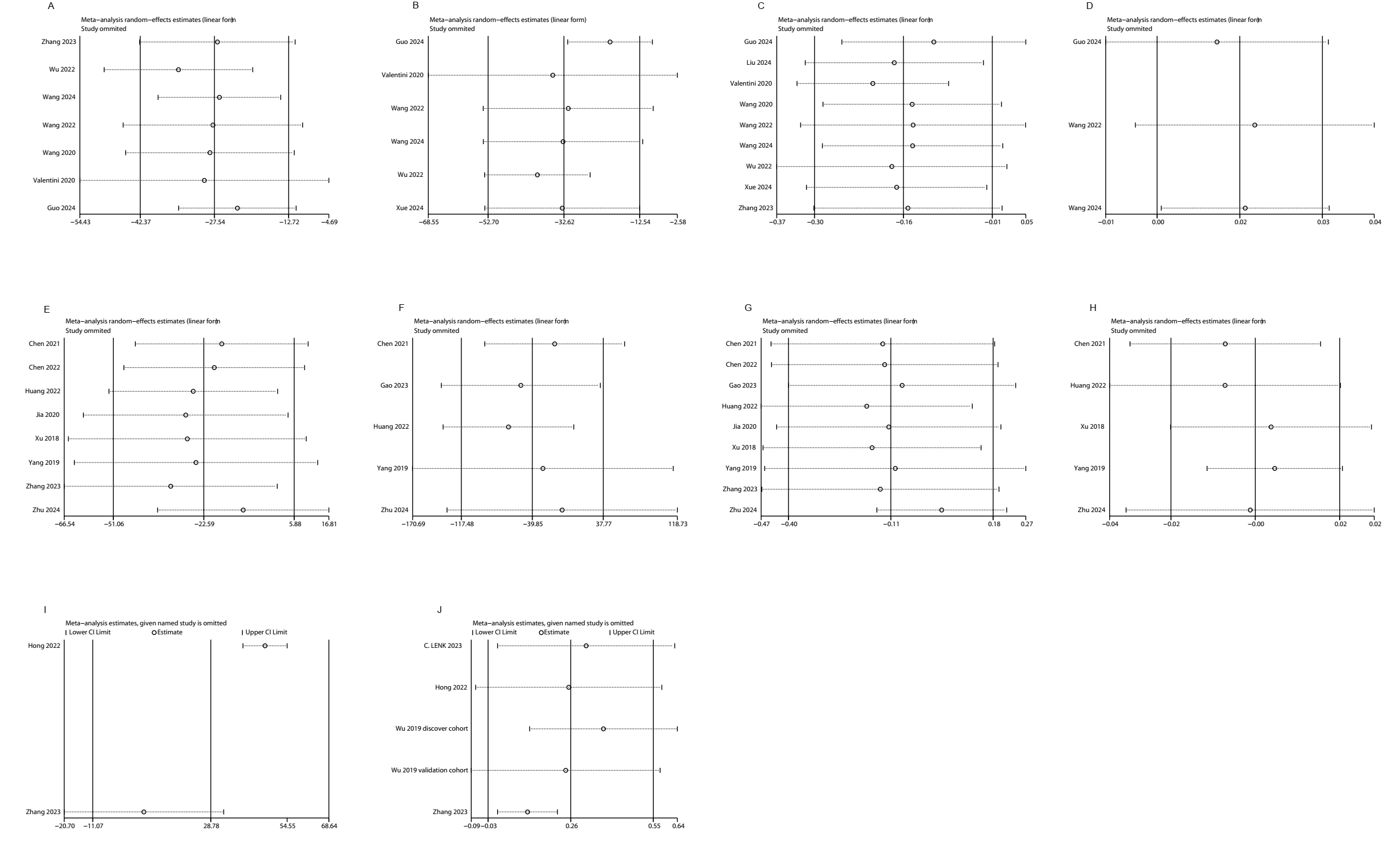


**Supplementary Figure 3:** Sensitivity analysis of gut and oral microbiota. A-D: Sensitivity analysis of gut microbiota; A: Chao1 index; B: Observed species; C: Shannon index; D: Simpson index; E-F: Sensitivity analysis of oral microbiota; E: Chao1 index; F: Observed species; G: Shannon index; H: Simpson index; I-J: Sensitivity analysis of respiratory tract microbiota, I: Chao1 index; J: Shannon index.


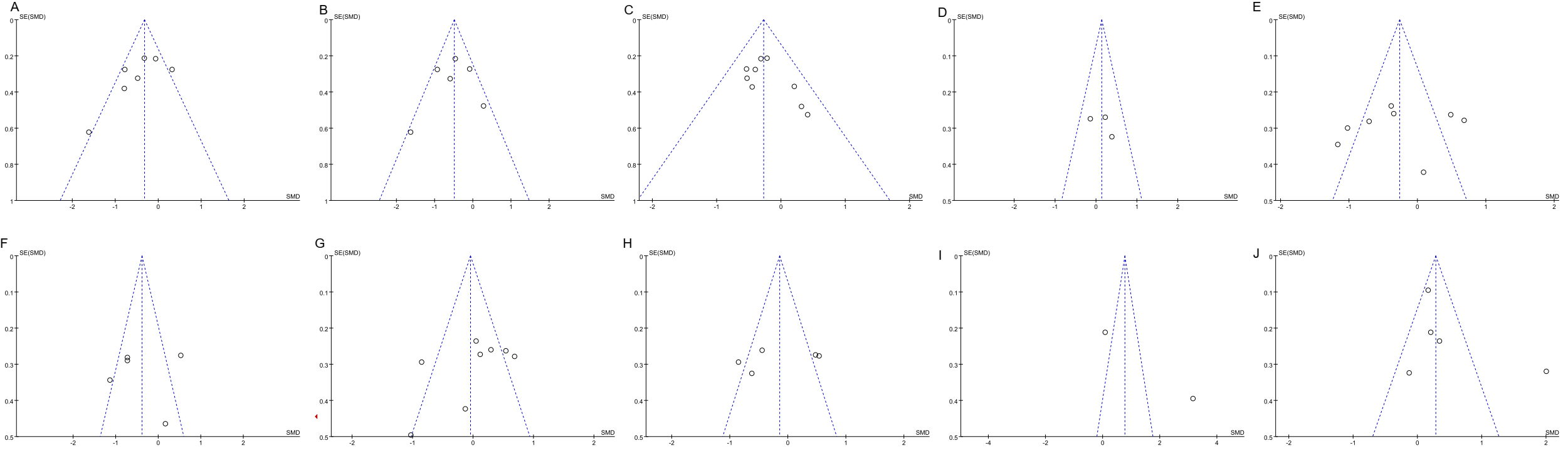


**Supplementary Figure 4:** Funnel plot of gut and oral microbiota. A-D: Funnel plot of gut microbiota; A: Chao1 index; B: Observed species; C: Shannon index; D: Simpson index; E-F: Funnel plot of oral microbiota; Chao1 index; F: Observed species; G: Shannon index; H: Simpson index; I-J: Funnel plot of respiratory tract microbiota, I: Chao1 index; J: Shannon index.

[1] F. Valentini, M. Evangelisti, M. Arpinelli, G. Di Nardo, M. Borro, M. Simmaco, and M.P. Villa, Gut microbiota composition in children with obstructive sleep apnoea syndrome: a pilot study. Sleep medicine 76 (2020) 140-147.

[2] L.J. Wang, C. Yang, Z.J. Dou, P.P. Wang, Z.X. Hu, and B. Wang, [Preliminary analysis of intestinal microflora in patients with different severity of OSAHS]. Zhonghua jie he he hu xi za zhi = Zhonghua jiehe he huxi zazhi = Chinese journal of tuberculosis and respiratory diseases 44 (2021) 543-549.

[3] F. Wang, Q. Liu, H. Wu, T. Tang, T. Zhao, and Z. Li, The dysbiosis gut microbiota induces the alternation of metabolism and imbalance of Th17/Treg in OSA patients. Archives of microbiology 204 (2022) 217.

[4] J. Wu, Y. Lu, X. Cai, Y. Chen, Z. Shen, and Q. Lyv, Gut microbiota dysbiosis in 4- to 6-year-old children with obstructive sleep apnea-hypopnea syndrome. Pediatric pulmonology 57 (2022) 2012-2022.

[5] C. Zhang, F. Chen, Y. Shen, Y. Chen, and J. Ma, Sleep apnea is associated with the increase of certain genera of Ruminococcaceae and Lachnospiraceae in the gut microbiome of hypertensive patients. Expert review of respiratory medicine 16 (2022) 1247-1256.

[6] W. Guo, L. Sun, H. Yue, X. Guo, L. Chen, J. Zhang, Z. Chen, Y. Wang, J. Wang, and W. Lei, Associations of Intermittent Hypoxia Burden with Gut Microbiota Dysbiosis in Adult Patients with Obstructive Sleep Apnea. Nature and science of sleep 16 (2024) 1483-1495.

[7] K.L. Liu, S.J. Xu, S.W. Chen, M.J. Zhang, N. Ye, and J. Li, Correlation Analysis of Characteristics of Intestinal Microbiota and Cytokine Levels in Patients with Obstructive Sleep Apnea-Hypopnea Syndrome. Nature and science of sleep 16 (2024) 1533-1544.

[8] P.P. Wang, L.J. Wang, Y.Q. Fan, Z.J. Dou, J.X. Han, and B. Wang, Analysis of the characteristics of intestinal microbiota in patients with different severity of obstructive sleep apnea. Scientific reports 14 (2024) 21552.

[9] X. Xue, Z. Zhao, L.B. Zhao, Y.H. Gao, W.H. Xu, W.M. Cai, S.H. Chen, T.J. Li, T.Y. Nie, D. Rui, Y. Ma, X.S. Qian, J.L. Lin, and L. Liu, Gut microbiota changes in healthy individuals, obstructive sleep apnea patients, and patients treated using continuous positive airway pressure: a whole-genome metagenomic analysis. Sleep & breathing = Schlaf & Atmung 29 (2024) 11.

[10] X. Chen, Y. Chen, M. Feng, X. Huang, C. Li, F. Han, Q. Zhang, and X. Gao, Altered Salivary Microbiota in Patients with Obstructive Sleep Apnea Comorbid Hypertension. Nature and science of sleep 14 (2022) 593-607.

[11] Y. Chen, X. Chen, X. Huang, Y. Duan, H. Gao, and X. Gao, Analysis of Salivary Microbiome and Its Association With Periodontitis in Patients With Obstructive Sleep Apnea. Frontiers in cellular and infection microbiology 11 (2021) 752475.

[12] P. Jia, J. Zou, S. Yin, F. Chen, H. Yi, and Q. Zhang, Analysis of the Salivary Microbiome in Obstructive Sleep Apnea Syndrome Patients. The Canadian journal of infectious diseases & medical microbiology = Journal canadien des maladies infectieuses et de la microbiologie medicale 2020 (2020) 6682020.

[13] X. Huang, X. Chen, X. Gong, Y. Xu, Z. Xu, and X. Gao, Characteristics of salivary microbiota in children with obstructive sleep apnea: A prospective study with polysomnography. Frontiers in cellular and infection microbiology 12 (2022) 945284.

[14] P.L.A.G.H. Chinese, Whole-genome metagenomic analysis of the oral microbiota in obstructive sleep apnea patients, European Nucleotide Archive, 2021.

[15] H. Xu, X. Li, X. Zheng, Y. Xia, Y. Fu, X. Li, Y. Qian, J. Zou, A. Zhao, J. Guan, M. Gu, H. Yi, W. Jia, and S. Yin, Pediatric Obstructive Sleep Apnea is Associated With Changes in the Oral Microbiome and Urinary Metabolomics Profile: A Pilot Study. Journal of clinical sleep medicine : JCSM : official publication of the American Academy of Sleep Medicine 14 (2018) 1559-1567.

[16] W. Yang, L. Shao, M. Heizhati, T. Wu, X. Yao, Y. Wang, L. Wang, and N. Li, Oropharyngeal Microbiome in Obstructive Sleep Apnea: Decreased Diversity and Abundance. Journal of clinical sleep medicine : JCSM : official publication of the American Academy of Sleep Medicine 15 (2019) 1777-1788.

[17] X. Zhang, X. Li, H. Xu, Z. Fu, F. Wang, W. Huang, K. Wu, C. Li, Y. Liu, J. Zou, H. Zhu, H. Yi, S. Kaiming, M. Gu, J. Guan, and S. Yin, Changes in the oral and nasal microbiota in pediatric obstructive sleep apnea. Journal of oral microbiology 15 (2023) 2182571.

[18] S. Zhu, and Z. Teng, [The characteristics of pharyngea microbiological in children with obstructive sleep apnea]. Lin chuang er bi yan hou tou jing wai ke za zhi = Journal of clinical otorhinolaryngology, head, and neck surgery 38 (2024) 1178-1182.

[19] C. Lenk, M.E. Messbacher, J. Abel, S.K. Mueller, K. Mantsopoulos, A.O. Gostian, M. Sievert, S. Wirtz, F. Marxreiter, J. Winkler, H. Iro, and M. Traxdorf, The influence of obstructive sleep apnea and continuous positive airway pressure on the nasal microbiome. European review for medical and pharmacological sciences 27 (2023) 2605-2618.

[20] S.N. Hong, K.J. Kim, M.G. Baek, H. Yi, S.H. Lee, D.Y. Kim, C.H. Lee, C. Shin, and C.S. Rhee, Association of obstructive sleep apnea severity with the composition of the upper airway microbiome. Journal of clinical sleep medicine : JCSM : official publication of the American Academy of Sleep Medicine 18 (2022) 505-515.

[21] B.G. Wu, I. Sulaiman, J. Wang, N. Shen, J.C. Clemente, Y. Li, R.J. Laumbach, S.E. Lu, I. Udasin, O. Le-Hoang, A. Perez, S. Alimokhtari, K. Black, M. Plietz, A. Twumasi, H. Sanders, P. Malecha, B. Kapoor, B.D. Scaglione, A. Wang, C. Blazoski, M.D. Weiden, D.M. Rapoport, D. Harrison, N. Chitkara, E. Vicente, J.M. Marin, J. Sunderram, I. Ayappa, and L.N. Segal, Severe Obstructive Sleep Apnea Is Associated with Alterations in the Nasal Microbiome and an Increase in Inflammation. American journal of respiratory and critical care medicine 199 (2019) 99-109.

[22] J. Zhu, M. Lu, Q. Jiao, Y. Sun, L. Liu, H. Ding, Y. Yu, and L. Pan, [Analysis of gut target microbiota and species difference in patients with obstructive sleep apnea based on 16S rRNA sequencing]. Nan Fang Yi Ke Da Xue Xue Bao 44 (2024) 146-155.

[23] C.Y. Ko, Q.Q. Liu, H.Z. Su, H.P. Zhang, J.M. Fan, J.H. Yang, A.K. Hu, Y.Q. Liu, D. Chou, and Y.M. Zeng, Gut microbiota in obstructive sleep apnea-hypopnea syndrome: disease-related dysbiosis and metabolic comorbidities. Clinical science (London, England : 1979) 133 (2019) 905-917.

[24] C.Y. Ko, A.K. Hu, D. Chou, L.M. Huang, H.Z. Su, F.R. Yan, X.B. Zhang, H.P. Zhang, and Y.M. Zeng, Analysis of oral microbiota in patients with obstructive sleep apnea-associated hypertension. Hypertension research : official journal of the Japanese Society of Hypertension 42 (2019) 1692-1700.
